# GRP78 regulates milk biosynthesis and the proliferation of bovinemammaryepithelial cells through the mTOR signaling pathway

**DOI:** 10.1186/s11658-019-0181-x

**Published:** 2019-10-22

**Authors:** Ying Liu, Xuemei Wang, Zhen Zhen, Yanbo Yu, Youwen Qiu, Wensheng Xiang

**Affiliations:** 1 The Key Laboratory of Dairy Science of Education Ministry, Heilongjiang Province, China; 2 HaiNanUniversity, Hainan Province, China

**Keywords:** Cyclin D1, GRP78, Bovine mammary epithelial cells, mTOR, SREBP-1c

## Abstract

**Background:**

Glucose-regulated protein 78 (GRP78) is a member of the HSP70 protein family and a key endoplasmic reticulum chaperone. It has been revealed to play important roles both in the maturation, folding and transport of proteins and in cellproliferation. However, its involvement in milk biosynthesis or the proliferation of bovine primary mammary epithelial cells (BMECs) has yet to be established.

**Methods:**

The expressions of GRP78 in BMECs stimulated with methionine, leucine, estrogen and prolactin were determined using western blotting and immunofluorescence assays. To explore the function of GRP78 in BMECs, the protein was overexpressed or knocked down, respectively using an overexpression vector or an siRNA mixture transfected into cells cultured in vitro. Flow cytometry was used to analyze cell proliferation and cell activity. The contents of lactose and triglyceride (TG) secreted from the treated BMECs were measured using lactose and TG assay kits, respectively. Western blotting analysis was used to measure the β-casein content and the protein levels of the signaling molecules known to be involved in milk biosynthesis and cell proliferation.

**Results:**

GRP78overexpression significantly stimulated milk protein and milk fat synthesis, enhanced cell proliferation, positively regulated the phosphorylation of mammalian target of rapamycin (mTOR), and increased the amount of protein of cyclinD1andsterol regulatory element-binding protein 1c (SREBP-1c). GRP78 knockdown after siRNA transfection had the opposite effects. We further found that GRP78 was located in the cytoplasm of BMECs, and that stimulating methionine, leucine, estrogen and prolactin expression led to a significant increase in the protein expression of GRP78 in BMECs.

**Conclusions:**

These data reveal that GRP78 is an important regulator of milk biosynthesis and the proliferation of BMECs through the mTOR signaling pathway.

## Background

Glucose-regulated protein 78 (GRP78, also known as the immunoglobulin binding protein BiP or HSPA5) is a member of the HSP70 protein family. It is a major endoplasmic reticulum (ER) chaperone with a molecular weight of 78 kDa. It can trigger the unfolded protein response (UPR), which is caused by ER-related stress [1–3]. GRP78 is composed of three domains: a peptide-binding domain, an ATPase domain, and a C-terminal domain [4], which contains the ER retention motif common to ER chaperones.

The UPR includes consists of three major branches, each of which comprises pathways mediated by protein kinase R-like ER kinase (PERK), inositol-requiring enzyme 1 (IRE1) and activating transcription factor 6 (ATF6)/branches: the protein kinase R-like ER kinase (PERK) pathway; the inositol-requiring enzyme 1 (IRE1) pathway; and the activating transcription factor 6 (ATF6) pathway [5]. GRP78 is known to be a key protein for cross-talk in the UPR [5].

Recent research into the mechanisms controlling the efficiency of milk biosynthesis in mammary glands has revealed the primary cellular signaling pathways leading to milk biosynthesis. Mammalian target of rapamycin (mTOR) has been described as a decisive mediator that integrates signals from growth factors and amino acids to regulate multiple biological processes related to milk protein synthesis and the proliferation of bovine primary mammary epithelial cells (BMECs) [6, 7]. Our previous study confirmed that some amino acids, such as methionine, leucine and lysine, and some hormones, such as prolactin and estrogen, can effectively activate mTOR, enhancing milk protein synthesis in BMECs [8–10].

Many studies have shown that mTOR functions as a central hub to control milk biosynthesis in and proliferation of BMECs [7, 9]. Sterol regulatory element-binding protein 1c (SREBP-1c) is a major transcription factor that regulates cellular fatty acid and triglyceride synthesis in BMECs [11, 12]. Cyclin D1 is a protein required for the progression from the G1 to the S and G2/M phases of the cell cycle [13]. In the signaling pathways related to milk biosynthesis and cell proliferation, mTOR is well known to regulate SREBP-1c and cyclin D1expression [14–16].

Previous reports have indicated that GRP78 may regulate the mTOR signaling pathway. It inhibits apoptosis by down regulating the AMPK-mediated inhibition of mTOR [17]. Its knockout was shown to suppress the activation of Akt/mTOR [18]. Cell surface-associatedGRP78might be associated with the activation of mTORC1 and mTORC2 signaling in prostate cancer cells [19]. Furthermore, low levels of its activity lead to the inhibition of rapamycin-sensitive mTORC1 [20].GRP78controls UPR and autophagy to regulate cell apoptosis, milk production during lactation, and subsequent mammary gland involution [21–23]. Thus, GRP78 is an upstream signaling molecule in the mTOR pathway, but its physiological role and molecular mechanismin milk biosynthesis still need further research.

In an earlier proteomics study (unpublished data), we found that GRP78 shows an association with milk biosynthesis and BMEC proliferation. We hypothesized that GRP78 might respond to extracellular stimuli to regulate milk biosynthesis and cell proliferation through the mTOR signaling pathway. In this study, we investigated the function of GRP78 in milk biosynthesis and cell proliferation, and further observed the expression and subcellular location of GRP78 in BMECs treated with methionine, leucine, estrogen or prolactin.

## Materials and methods

### Primary cellculture and treatment

Primary BMECs were obtained from healthy mid-lactation Holstein cow mammary tissues purchased on a market in Harbin, China. Cells were cultured and purified as previously reported [24, 25]. The purity of the cells was evaluated by observing the microscopic morphology and the immune fluorescence of the expression of cytokeratin 18 (CK18) in the cells, as previously reported [24]. Cells were normally cultured in culture bottles containing Dulbecco’s modified Eagle’s medium (DMEM) and Ham’s F-12 in a 1:1 mixture (DF-12 medium; SH30023.01B; HyClone, Thermo Fisher Scientific) with 15% fetal bovine serum (FBS) and 1% streptomycin and penicillin. The culture conditions were 37 °C in a humidified atmosphere containing 5% (v/v) CO_2_.To investigate the effects of methionine, leucine, estrogen or prolactin stimulation on GRP78 protein expression, cells were incubated in six-well plates containing DF-12 medium without FBS and treated with methionine (0.6 mM), leucine (0.6 mM), estrogen (27.2 ng/ml),or prolactin(50 μg/ml) for 24 h [16]. After the treatments, the cells and supernatants were separately collected for detection.

### Immunofluorescence to detect GRP78 expression

Cells were cultured on cover slips in 6-well plates for2 days for immune fluorescence staining. Then, they were fixed in 4% paraformaldehyde for 30 min and washed twice in Tris-buffered saline (TBS). To prevent non-specific protein binding, treated cells were incubated with 5% BSA at 37 °C. After two washes with TBS, the cells were treated with 0.2% Triton X-100 for 30 min and then incubated overnight at 4 °C with primary antibodies targeting CK18 (1:200, 10,830–1-AP, Proteintech) or GRP78 (1:200, 11,587–1-AP, Proteintech). Next, the cells were washed twice with TBS and incubated with mouse anti-rabbit IgM/AlexaFluor 488 antibody (bs-0369 M-AF488;BIOSS) or goat anti-mouse IgG/AlexaFluor 647 antibody (bs-0296G-AF647;BIOSS) at 37 °C for 30 min. The cells were then washed twice in TBS and dyed using DAPI (28718–90-3, Sigma Aldrich) for15 min. Images of the stained cells were captured with the GE DeltaVision OMX SR system. For quantitative analysis of the images, the AIOD (area-inter-grated optical density) of GRP78 per cell was calculated using ImageJ. Ten cells were analyzed for each sample.

### Vector construction and transfection

The *Bostaurus* GRP78/HSPA5coding DNA sequence (CDS; NM_001075148.1) was amplified at the Beijing Genomics Institute and was cloned into apcDNA3.1 vector (Addgene, 52,535, Biovector). Using Lipofectamine 3000(L3000–015;Thermo Fisher Scientific),the plasmids were transfected into BMECs according to the manufacturer’s instructions. Cells transfected with empty vector served as a negative control. Cells were collected 48 h after transfection and used for subsequent experiments.

### siRNA transfection

AGRP78 siRNA pool with three siRNAs targeting different portions of the *GRP78* mRNA sequence was created and manufactured by GenePharma. Scramble siRNA oligonucleotides, which served as a negative control (siRNA-NC), were produced by GenePharma. They were designed to have no homology with any bovine gene. The sequences were: si-GRP78–1, 5′-GGGAAAGAAGGUUACUCAUTT-3′; si-GRP78–2, 5′-AUCCAUUGAUAAUGGUGUCUUTT-3′; si-GRP78–3, 5′-GCGCAUCGACACAAGAAAUTT-3′; and siRNA-NC UUGUACUACACAAAAGUACUG.

Using Lipofectamine 3000, the cells were transfected with either the GRP78 siRNA pool or siRNA-NC according to the manufacturer’s protocol. The efficiency of transfection with this siRNA pool was verified through western blotting analysis of the expression of GRP78.At 24 h post-treatment, cells were collected for detection.

### Western blotting

Western blotting was performed as previously described [24]. Briefly, cells were rinsed in cold phosphate-buffered saline (PBS) and lysed with a lysis buffer (Beyotime) at 4 °C. After centrifugation, 30-μg protein samples were subjected to SDS-PAGE, transferred to nitrocellulose membranes, blocked using 5% skim milk dissolved in TBST, and incubated with primary antibodies overnight at 4 °C. The membranes were cleaned with TBST, then incubated with horseradish peroxidase-conjugated anti-rabbit IgG (ZSGB-Bio) for 1 h at 37 °C. Enhanced chemiluminescence (ECL) substrate (Sage Brightness) was used to detect the horseradish peroxidase. The primary antibodies were: GRP78 (1:500, 11,587–1-AP;Proteintech), mTOR (1:500, ab2833;Abcam), p-mTOR (Ser2448; 1:1000, #2971;Cell Signaling Technology), SREBP-1c (1:500, 14,088–1-AP;Proteintech), cyclin D1 (1:500, 60,186–1-Ig;Proteintech), β-casein (1:1000, bs-0813R;BIOSS), and β-actin (1:1000, M1501;HaiGene).

### Measurement of β-casein, lactose and triglyceridelevels

The levels of β-casein proteinin BMECs were determined via western blotting analysis. Triglyceride and lactose amounts secreted into the culture medium by BMECs were respectively detected using a TG GPO-POD Assay Kit (ApplygenTech) and Lactose Assay Kit (Megazyme), according to the manufacturer’s protocol.

### Analysis of cell number and cell cycle progression

Cell number was automatically calculated using an automatic cell counter (Model DT CASY, Scharfe System GmbH) according to the manufacturer’s protocol and our previous report [24]. Cell cycle progression was determined using the method described in our previous report [16]. Briefly, cells were washed with cold PBS, trypsinized, and collected by centrifugation. Then, the cells were fixed with cold 75% ethanol at 4 °C overnight, washed 3 times with PBS, and then were re-suspended in PBS containing 5 μg/ml propidium iodide (Pharmingen) and 0.1 mg/ml RNase A.Finally,BMECs were incubated for 15 min in the dark at room temperature and then analyzed viaflow cytometry using a Guava EasyCyte HT system (Merck Millipore). The proportion (%) of cells in each cell cycle phase was calculated based on the flow cytometry results.

### Statistical analysis

The experimental data are presented as the means ±standard error for each group from three independent experiments. Statistical analyses were perform edusing Student’s t test orone-way ANOVA with Prism 5 software (SPSS, Inc.). Tukey’s post hoc test was used to analyze the differences between the means of individual groups. A value of *p* < 0.05 or *p* < 0.01 was considered statistically significant.

## Results

### GRP78 is involved in milk biosynthesis and cell proliferation

**Fig. 1:**
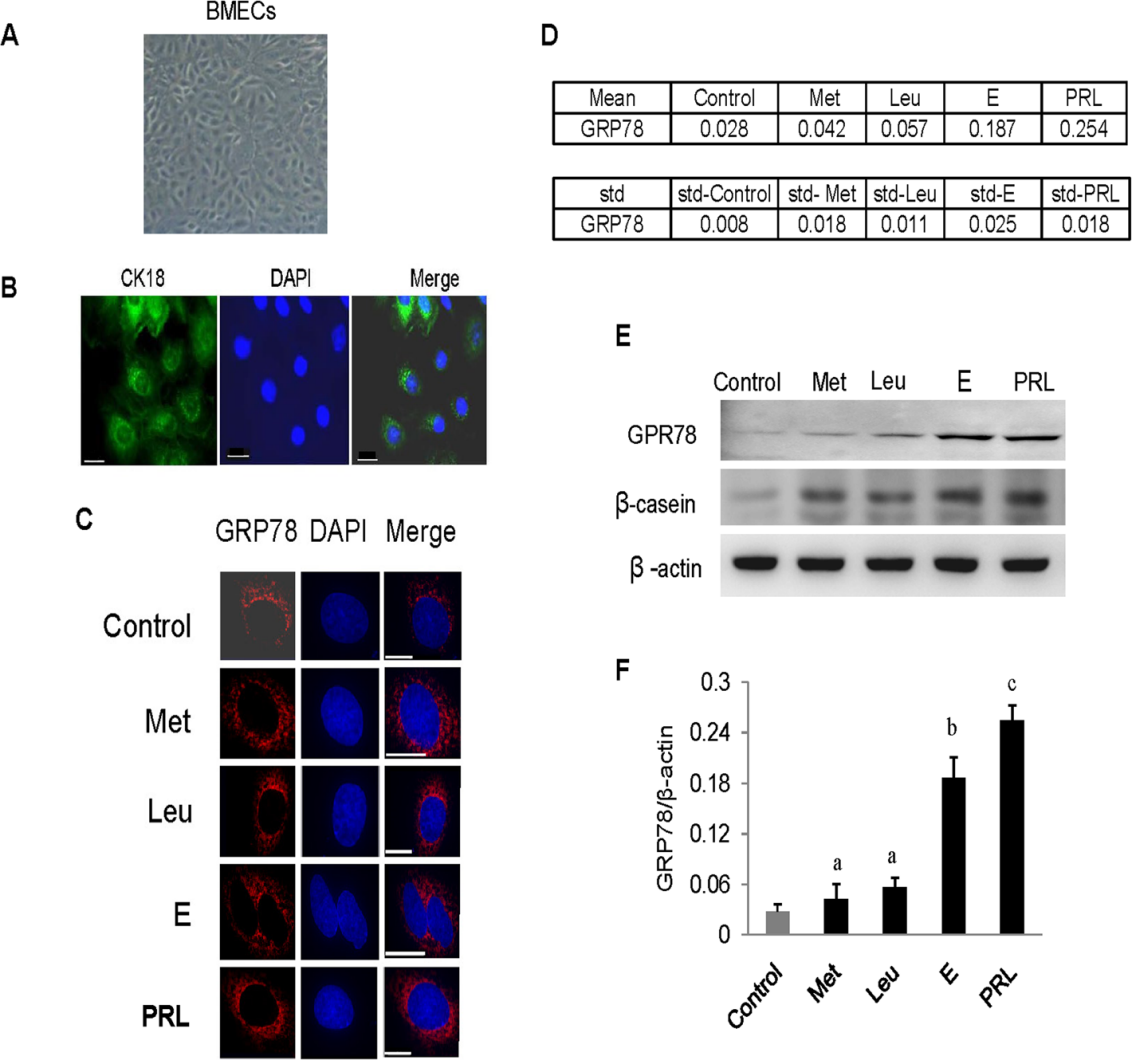
Certain amino acids and hormones trigger GRP78 expression. **a** Microscopic morphologyof the purified cells. Scale bar: 75 μm. **b** Detection of CK18 in BMECs. CK18 (green), DAPI (blue), scale bar: 25 μm.**c** Subcellular localization of GRP78 in cells treated with the essential amino acids Met and Leu and the hormones estrogen (**e**) or prolactin (PRL). Nuclei (blue), GRP78(red), scale bar: 10 μm. **d** AIOD of GRP78 expression per cell from (**b**) was analyzed using ImageJ. Ten cells were analyzed per sample. The AIOD of the control was set as 1.00 for clarity. **e** The protein levels of GRP78 and β-casein in the cells were determined using western blotting after treatments with methionine (Met), leucine (Leu), estrogen (**e**) or prolactin (PRL); β-actin served as the loading control. **f** The relative fold changes in the protein levels of GRP78 (protein/β-actin) in (**e**) were quantified with grayscale scanning. The data represent the means±SE from three independent experiments. Values with different superscripted lowercase letters indicate significant difference (*p* < 0.05)

In this study, BMECs were successfully purified from the mammary glands of dairy cows and identified based on their microscopic morphology and immunofluorescence. The mixed fibroblasts containing long fibers were removed viatrypsin digestion. The purified cells exhibited essentially the same round or oval cell morphology (Fig. 1a). Their purity was verified using immunofluorescence observation. Nearly all the cells in the field of vision exhibited strong positive staining for CK18 (Fig. 1b), confirming that the purified cells from the mammary gland were epithelial cells.

To show that GRP78 was associated with milk biosynthesis and cell proliferation, we investigated whether GRP78 expression changed in response to extracellular stimuli. Immunofluorescence results showed that GRP78 localized to the cytoplasm, and that amino acids methionine, leucine, estrogen or prolactin stimulated an increase in its expression (Fig. 1c and d).

To determine the quantitative effects of these stimuli on GRP78 expression, we used western blotting to measure the amount of GRP78 and β-casein protein after the treatments. The β-casein protein contents significantly increased (Fig. 1e), showing the regulatory effects of the stimuli, which is consistent with our previous study [14]. We found that the protein levels of GRP78 significantly increased up on hormone stimulation (estrogen or prolactin), whereas the amino acids (methionine and leucine) had weaker effects (Fig. 1e and f). These data reveal that GRP78 is regulated by extracellular stimuli, such as hormones and amino acids, suggesting that it might be involved in amino acid- or hormone-stimulated milk biosynthesis and cell proliferation.

### GRP78 knockdown decreases milk biosynthesis and cell proliferation

**Fig. 2:**
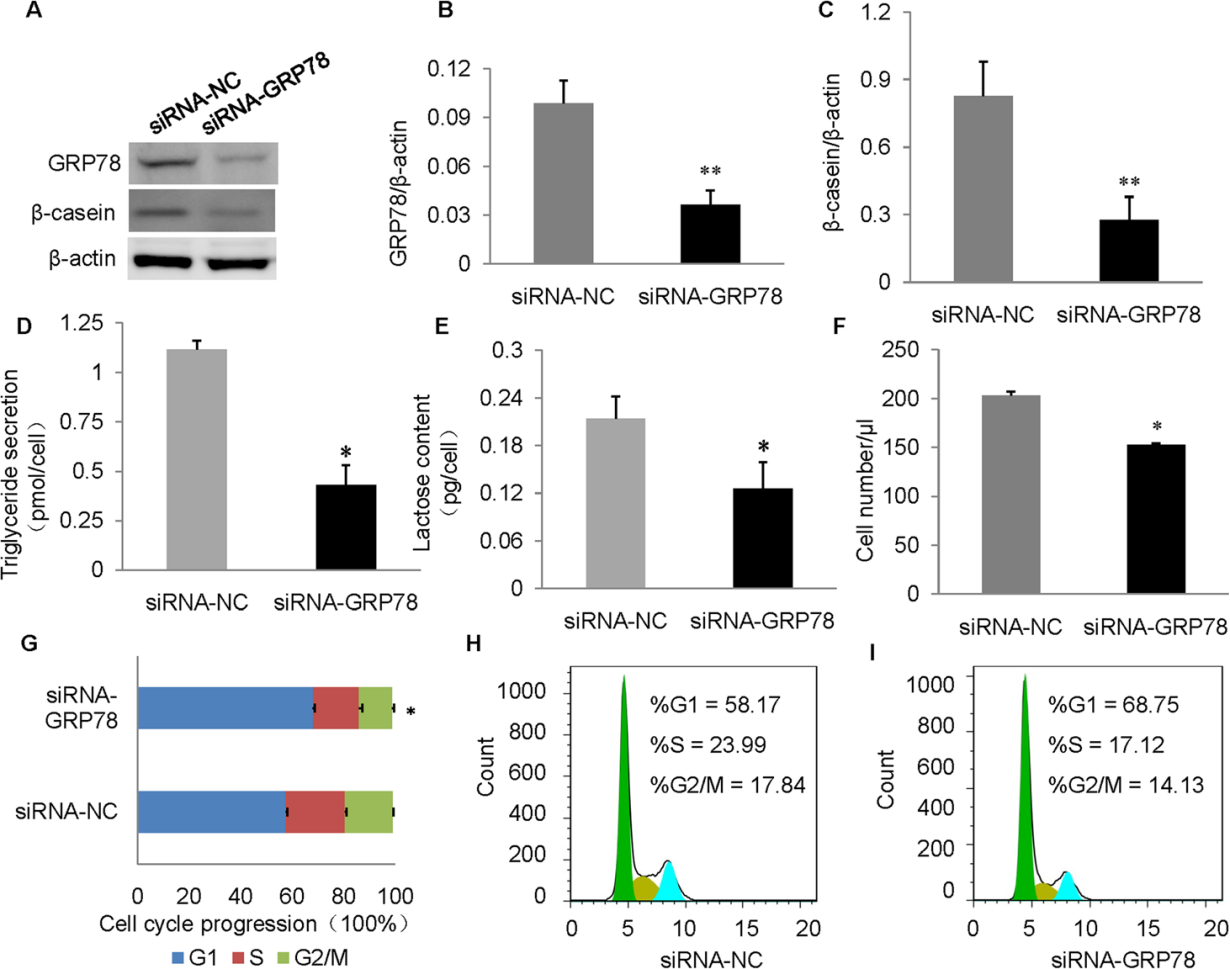
RNAi of *GRP78* suppresses milk biosynthesis and cell proliferation. **a** Western blotting analysis of GRP78 and β-casein in BMECs transfected with siRNA targeting GRP78. Cells transfected with scramble siRNA were used as a negative control (siRNA-NC). **b** and **c** Relative fold changes in protein levels (proteins/β-actin)of GRP78 (**b**) and β-casein (**c**) determined via western blotting and quantified using grayscale scanning. **d** Triglyceride (TG) contents in the culture medium. **e** Lactose contents in the culture medium. **f** Cell numbers measured using a cell counter. **g**, **h** and **i** Cell cycle transition measured (**g**) and analyzed (**h** and **i**) via flow cytometry. Data represent the means ± SE from three independent experiments. *p < 0.05;**p < 0.01

To investigate whether GRP78 plays a regulatory role in milk biosynthesis and cell proliferation, we knocked down GRP78 by transfecting cells with siRNA targeting GRP78. The amount of GRP78 was dropped by ~ 70% in the siRNA transfection group, as shown in western blotting analysis (Fig. 2a and b). Knockdown of GRP78 significantly decreased the β-casein levels (Fig. 2a and c), triglyceride levels (Fig. 2d) and lactose secretion level (Fig. 2e) in the cells, compared to the siRNA NC group. GRP78 knockdown markedly decreased the cell number (Fig. 2f) and the percentage of cells in the S and G2/M phases, while significantly increasing the percentage of cells in G1 phase (Fig. 2g and h). These results suggest that GRP78 positively regulates milk biosynthesis and cell proliferation.

### GRP78 overexpression increases milk biosynthesis and cell proliferation

**Fig. 3:**
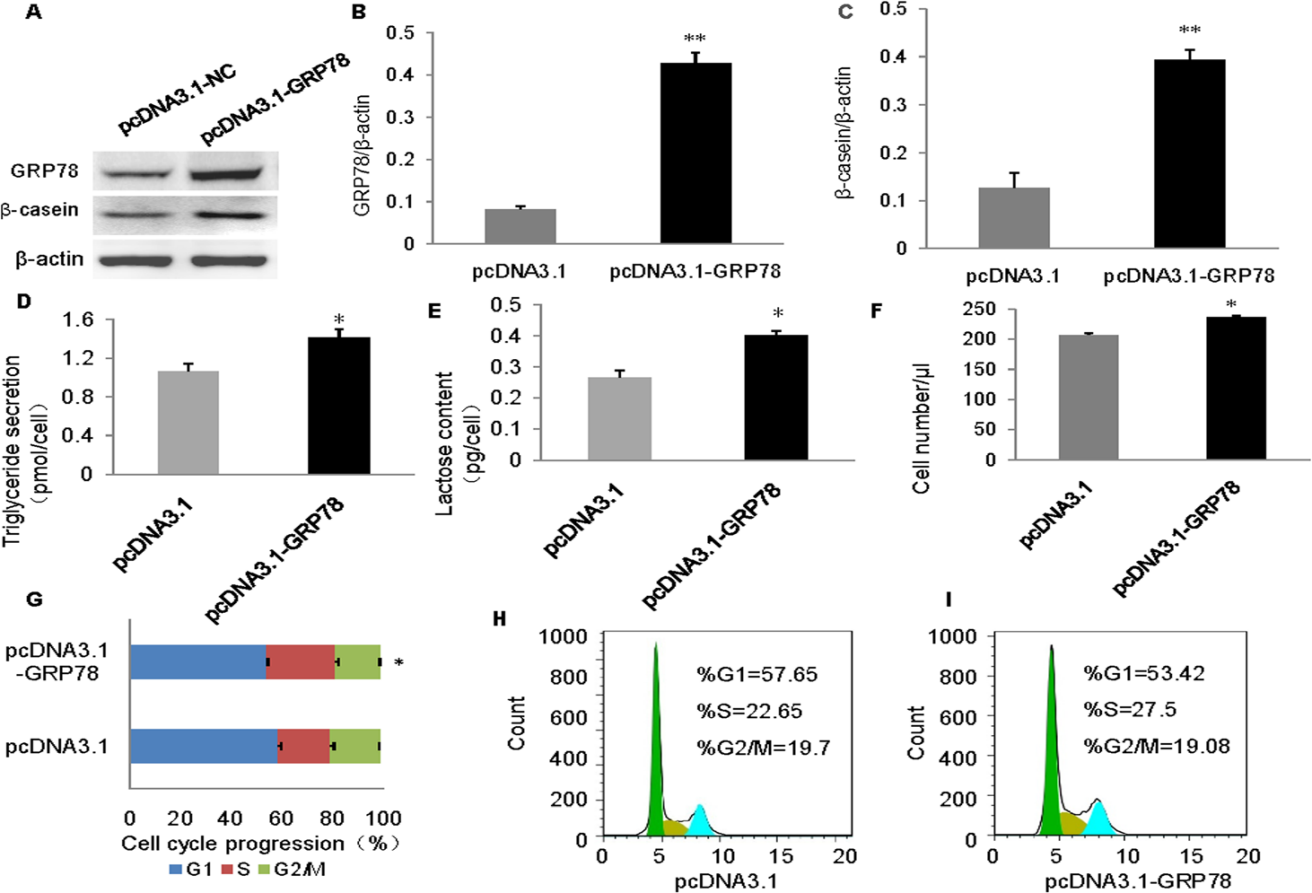
Overexpression of GRP78 promotes milk biosynthesis and cell proliferation. **a** Western blot analysis of GRP78 and β-casein in BMECs transfected with pcDNA3.1-GRP78 vector. Cells transfected with the empty vector (pcDNA3.1) were used as negative controls. **b** and **c** Relative fold change of protein levels (proteins/β-actin) of GRP78 (**b**) and β-casein (**c**) determined via western blotting and quantified using grayscale scanning. **d** Triglyceride (TG)contents in the culture medium. **e** Lactose contents in the culture medium. **f** Cell numbers were measured using a cell counter. **g**, **h** and **i** Cell cycle transition measured (**g**) and analyzed (**h** and **i**) via flow cytometry. Data represent the means ± SEfrom three independent experiments. *p < 0.05;**p < 0.01

To further prove that GRP78 positively regulates milk biosynthesis and cell proliferation, we constructed the pcDNA3.1-GRP78 vector and transfected it into the cultured cells. This led to a significant increase in the amount of GRP78 protein (Fig. 3a and b). Over expression of GRP78 significantly increased the β-casein levels (Fig. 3a and c), triglyceride levels (Fig. 3d) and lactose secretion level (Fig. 3e) compared to the empty vector group. It also markedly increased cell number (Fig. 3f) and the percentage of cells in the S and G2/M phases, while significantly decreasing the percentage of cells in the G1 phase (Fig. 3g and h). These results provide further evidence that GRP78 is a positive regulator of milk biosynthesis and cell proliferation.

### GRP78 positively regulates mTOR signaling

**Fig. 4:**
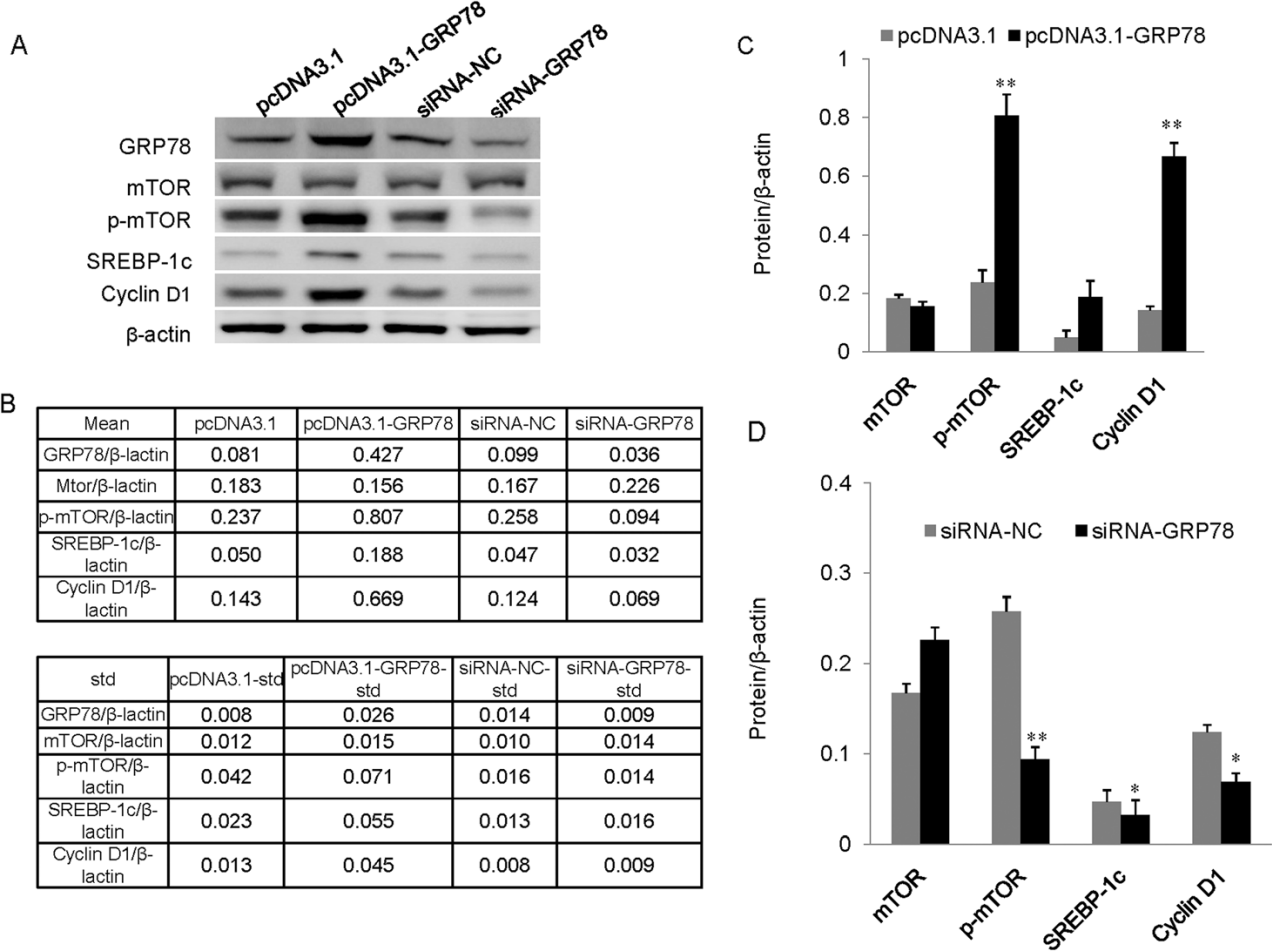
GRP78 is a positive regulator of the mTOR signaling pathway. **a** BMECs were transfected with pcDNA3.1-GRP78 vector or GRP78siRNA. Cells transfected with the empty vector (pcDNA3.1) or scramble siRNA were used as negative controls. The indicated protein levels were detected using western blotting analysis. β-actin served as a loading control. **b** Relative fold changes inGRP78 protein levels (protein/β-actin) in (**a**) were quantified using grayscale scanning. **c** and **d** Relative fold changes of indicated protein levels (protein/β-actin) were quantified using gray scale scanning after over expression (**c**) or silencing (**d**) of GRP78

To investigate the mechanism through which GRP78 regulates milk biosynthesis and cell proliferation, it was over expressed or knocked down in cells to reveal its impact on the expression of signaling molecules responsible for milk biosynthesis and cell proliferation. GRP78 over expression and knockdown in the cells were confirmed and signaling molecule expressions were determined using western blotting analysis (Fig. 4a and b). GRP78 over expression significantly upregulated the protein levels of p-mTOR, cyclin D1 and SREBP-1c compared to the empty vectorgroup (Fig. 4a and c). GRP78 knockdown through siRNA transfection had opposite effects (Fig. 4a and d). The mTOR expression level was unchanged in cells after GRP78 was over expressed or knocked down, suggesting a balance between mTOR and its phosphorylation form in cells, which is consistent with the results of our previous studies [8–10]. These results reveal that GRP78 positively regulates milk biosynthesis and cell proliferation via the mTOR or SREBP-1c or cyclin D1 signaling pathway.

## Discussion

The aim of this study was to confirm the importance of GRP78 in milk biosynthesis and in the proliferation of BMECs. mTOR, cyclin D1and SREBP-1c are all critical signaling molecules for milk biosynthesis and cell proliferation, with mTOR acting as a central hub. Increased GRP78 expression was shown to enhance the levels of p-mTOR, cyclin D1 and SREBP-1c, thereby increasing the biosynthesis of milk components and BMEC proliferation. To the best of our knowledge, this is the first report ofGRP78 being involved in milk biosynthesis and cell proliferation through the mTOR signaling pathway.

Some hormones (including estrogen and prolactin) can stimulate the development of the mammary gland and milk biosynthesis [26]. Through estrogen receptor α (ERα), estrogen can induce rapid UPR activation, thereby increasing the expression of GRP78 [27–29]. GRP78 protein was previously shown to be upregulated in prolactin-treated mouse MECs [23]. Our data are consistent with these previous reports, also showing that estrogen and prolactin positively influence the expression of GRP78.

The molecular mechanism through which hormones stimulate the expression of GRP78 is still largely unknown. Previous studies have found that estrogen triggers the expression of GRP78 through phosphoinositide 3-kinase (PI3K) signaling [30]. Others have observed that cell-surface GRP78 is a multi-functional receptor that can form complexes with PI3K as a regulator of the PI3K/Akt signaling pathway to exert its function [19]. How PI3K is involved in hormone-stimulated GRP78 expression and function requires further research.

Amino acids serve not only as components for protein synthesis, but also as signaling molecules that regulate milk biosynthesis through activation of the mTOR signaling pathway [31, 32]. We observed that the expression of GRP78 increased after methionine and leucine stimulation, although their effects were far less than those of estrogen and prolactin.

It is currently unclear how amino acids stimulate GRP78 expression. Previous reports have shown that amino acids activate the mTOR signaling pathway through the expression of certain G protein-coupled receptors (GPCR) and PI3K [33, 34]. Our findings support the hypothesis that amino acids might regulate GRP78 expression through GPCR-PI3K signaling.

mTOR is a crucial kinase that regulates various cell functions, such as cell cycle progression, cell proliferation, and protein and fat synthesis [35, 36]. mTOR regulates many downstream targets, including S6K1, 4EBP1, SREBP-1cand cyclin D1,to activate milk biosynthesis and cell proliferation [37, 38]. Our gene function studies showed that GRP78 could positively regulate signaling molecules such as mTOR, cyclin D1 and SREBP-1c.However, the molecular mechanism through which GRP78 regulates mTOR signaling is still unknown. In our previous studies, we found that amino acids and hormones can stimulate mTOR signaling via NF-κB1 activation [26]. Whether GRP78 activates mTOR signaling directly in the cytoplasm or throughNF-κB1-mediated transcriptional activation requires further study.

It is interesting that GRP78,which can trigger UPR, can positively regulate the synthesis of milk components,cell proliferation and the corresponding signaling pathways. The role and underlying molecular mechanisms of UPR in amino acid- and hormone-stimulated GRP78 expression and GRP78-stimulated mTOR signaling and cell homeostasis is still largely unknown. Previous reports have pointed out that UPR and GRP78 can be induced by the spliced X-box binding protein 1 (XBP1)and ATF6 [39, 40], and that GRP78 can clear unfolded stress-inducing protein to reinstate ER homeostasis and eukaryotic initiation factor2α (eIF2α) expression and protein synthesis [41, 42]. Thus, we speculate that certain amino acids and hormones, including those tested, might stimulateGRP78 expression via activation of XBP1 and ATF6, and that GRP78 might function through the UPR and eIF2α expression. Previous reports have also pointed out that mTOR can inhibit GRP78 expression [43, 44], suggesting thatthe relationship between GRP78 and mTOR might be bidirectional rather than straight.

## Conclusions

Our results show that GRP78 is a key positive regulator of milk biosynthesis and the proliferation of BMECs. It was found to respond to extracellular stimuli, such as amino acids and hormones, and to activate mTOR signaling, leading to milk biosynthesis and increased cell proliferation. The signaling pathway related to GRP78 expression and function requires further research.

## Data Availability

All data generated or analyzed during this study are included in this published article and its supplementary information files.

## Abbreviations

ATF6: Activating transcription factor 6
CDS: Coding DNA sequence
CK 18: Cytokeratin 18
DMEF: Dulbecco’s modified Eagle’s medium
ECL: Enhanced chemiluminescence
ER: Endoplasmic reticulum
GPCR: G protein-coupled receptors
GRP78: Glucose-regulated protein 78
HRP: Horseradish peroxidase
IRE1: Inositol-requiring enzyme 1
MECs: Mammary epithelial cells
mTOR: Mammalian target of rapamycin
PERK: Protein kinase R-like ER kinase
PI3K: Phosphoinositide 3-kinase
SREBP-1c: Sterol regulatory element-binding protein 1c
TBS: Tris-buffered saline
UPR: Unfolded protein response
